# Urological Complications Associated With Pyeloureterostomy Without Ipsilateral Nephrectomy in Renal Transplant Recipients

**DOI:** 10.3389/ti.2021.10213

**Published:** 2022-01-18

**Authors:** Hernani M. Neto, Helio Tedesco Silva Junior, José M. Pestana, Renato D. Foresto, Wilson F. Aguiar

**Affiliations:** ^1^ Department of Urology, Federal University of Sao Paulo, Sao Paulo, Brazil; ^2^ Hospital do Rim (HRim), Sao Paulo, Brazil; ^3^ Department of Nephrology, Federal University of Sao Paulo, Sao Paulo, Brazil

**Keywords:** kidney transplant recipients, pyeloureterostomy, nephrectomy, urinary complications, urinary leak, ureteral stenosis

## Abstract

**Background:** The implications of ligating the native ureter without ipsilateral nephrectomy after primary kidney transplant pyeloureterostomy (PU) have been described previously.

**Methods:** This single-center retrospective cohort study including 4,215 kidney transplants performed between February 2010 and December 2014, analyzed urological complications following primary (P-PU) and secondary (S-PU) pyeloureterostomy used to treat urological leaks (UL-PU) and ureteral stenosis (US-PU) without concomitant ipsilateral nephrectomy, in a large cohort of patients.

**Results:** There were 495 (11.7%) pyeloureterostomy with native ureter ligation without nephrectomy, 409 P-PU (82.6%) and 86 S-PU (17.4%), of which 76 were UL-PU and 10 were US-PU. The median follow-up was 33.8 months. The incidence of native ipsilateral kidney complications requiring nephrectomy was 2.02% (n = 10). Urinary leak was diagnosed in 3.6% of patients after P-UP and 9.2% after UL-PU. Ureteral stenosis was diagnosed in 1.7% of patients after P-UP, 3.9% after UL-PU and 10% after US-PU.

**Conclusion:** This cohort analysis suggests that native ureter ligation during pyeloureterostomy without native nephrectomy is associated with low incidence of clinically indicated ipsilateral native nephrectomy. Caution and awareness should be emphasized in patients with history of ADPKD and neurogenic augmented bladders.

## Graphical Abstract

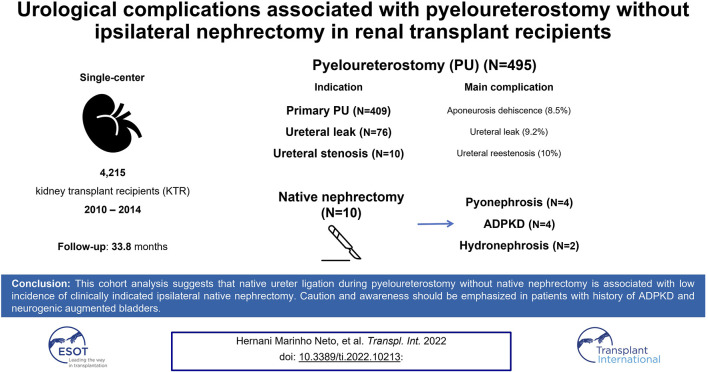

## Introduction

Classical techniques for urinary tract reconstruction during a kidney transplant surgery include reimplantation of the kidney donor ureter with the recipient´s bladder (ureteroneocystostomy) or with the recipient’s native ureter (pyeloureterostomy or ureteroureterostomy). While both techniques show similar urological complication rates, most transplant centers initially opt for a ureteroneocystostomy using the Lich-Gregoir technique (1–3), deferring the use of ureteroureterostomy, usually without ipsilateral nephrectomy, as a secondary option in case of complications in the ureteroneocystostomy anastomosis, such as urinary leak and ureteral stenosis (3–10). Although some reports have shown that the native ureter ligation without nephrectomy is safe (3–8), this technique may cause hydronephrosis, primarily in patients with significant residual diuresis, and eventually discomfort or lumbar pain.

As a primary objective, we evaluated the risk of future nephrectomy in these patients, and the secondary objective was to assess other urological complications with the need for surgical intervention.

## Methods

This was a single-center, retrospective cohort study that included data from the electronic records of all patients who underwent kidney transplantation from February 2010 to December 2014 at Hospital do Rim, Brazil. The local Ethics Committee approved this study. Patients with missing demographic or surgical data were excluded. For this analysis, only urological complications that required new surgical procedures were analyzed. Continuous variables were presented as mean and standard deviation, and categorical variables were presented as frequencies.

Our routine reimplantation technique is a Lich-Gregoir procedure without stenting, saving the pyeloureterostomy (PU) for three key indications: 1. difficult access to the bladder; 2. doubtful graft ureter viability; 3. as a secondary anastomosis method to treat ureteroneocystostomy complications (urine leak or stenosis). All cases, the PU included a simple proximal native ureter ligation, leaving the obstructed kidney *in situ*. The anastomosis between the renal pelvis and the spatulated distal native ureter is performed in an end-to -end technique using running 6.0 polydioxanone sutures (PDS^®^ II). A double-J ureteral stent (6 fr × 18 cm) was left for 28 days and an indwelling urinary 20 fr Foley catheter for 7 days.

## Results

### Demographic Characteristics and Prevalence of Pyeloureterostomy

From December 2010 to February 2014, a total of 4,215 kidney transplants were performed in our institution. We excluded 264 (6.3%) patients due to incomplete data. Of the remaining 3,951 transplanted patients, 2,903 (73.5%) received a kidney from a deceased donor and 1,048 (26.5%) from a living donor. Of them, 495 (12.5%) patients underwent pyeloureterostomy, 409 (10.3%) as a primary procedure performed at the time of the transplant (P-PU) and 86 (2.2%) as a secondary technique to treat urinary leak (UL-PU, n = 76) or ureteral stenosis (US-PU, n = 10). Demographic characteristics of the study population are described in Table 1 **.**

**TABLE 1:** Demographic characteristics of the study population.

Variable, n (%)	Total (n = 495)	P-PU (n = 409)	UL-PU (n = 76)	US-PU (n = 10)
Recipients				
Age, years	48.7 ± 13.2	49.8 ± 12.8	42.8 ± 13.9	46.4 ± 11.8
Gender, male	332 (67)	277 (68.2)	50 (64.9)	5 (50)
Ethnicity, white	269 (54.3)	233 (57.3)	39 (50.6)	7 (70)
CKD etiology				
Undetermined	201 (40.6)	161 (39.2)	35 (46)	5 (50)
Hypertension	83 (16.7)	71 (17.3)	10 (13.1)	2 (20)
Diabetes Mellitus	58 (11.7)	49 (11.9)	8 (10.5)	1 (10)
Glomerulopathy	69 (14.1)	58 (14.1)	11 (14.4)	0
ADPKD	33 (6.6)	28 (6.8)	3 (3.9)	2 (20)
Neurogenic bladder	11 (2.2)	9 (2.2)	2 (2.5)	0
Other	40 (8.2)	33 (8.0)	7 (9.2)	0
BMI, Kg/m^2^	25 ± 4.3	25 ± 4.5	23 ± 5.6	23 ± 4.5
Diabetes Mellitus	82 (16.6)	68 (16.7)	13 (16.8)	1 (10)
Hemodialysis	443 (89.4)	372 (90.9)	65 (85.5)	6 (60)
Dialysis time, months	73,4 ± 60.3	80,0 ± 61.2	38 ± 32.4	46.8 ± 28.2
Residual diuresis, mL/day	221 ± 431	164 ± 347	524 ± 649	360 ± 337
Donor				
Deceased	429 (86.6)	367 (89.7)	52 (68.4)	10 (100)
Living	66 (13.3)	42 (8.6)	24 (31.6)	0

BMI, body mass index; CKD, chronic Kidney disease ADPKD, autosomal dominant polycystic kidney disease.

### Urological Complications

#### Primary Pyeloureterostomy

Of 409 P-PU, 367 were performed in deceased (89.7%) and 42 (10.3%) in living donor kidney transplant recipients. All these cases were performed for two reasons: 1. difficult access to the bladder; 2. doubtful graft ureter viability.

As indicated in Table 2, urinary leakage occurred in 15 patients (3.6%) between 2 and 45 days after the P-PU. In 13 patients (87%), the pyeloureterostomy was remade over a double-J catheter, and five required two surgical procedures, including a protective nephrostomy. One of these patients developed a deep surgical site infection requiring graft nephrectomy 56 days after transplantation. Finally, one (6.6%) patient was treated with a single suture stitch, and another one (6.6%) was treated conservatively by retrograde insertion of a double-J ureteral catheter and an indwelling urinary catheter.

**TABLE 2:** Surgical complications.

Primary pyeloureterostomy	n = 409
Total, n (%)	107 (26.1)
Aponeurosis dehiscence	35 (8.5)
Isolated	23
With skin dehiscence	4
With surgical site infection	3
With hematoma	3
With internal hernia	1
With skin dehiscence and surgical site infection	1
Ureteral leak	15 (3.6)
Isolated	11
With hematoma	1
With surgical site infection	1
With aponeurosis dehiscence and hematoma	1
With aponeurosis dehiscence and surgical site infection	1
Perigraft hematoma	12 (2.9)
Surgical site infection	11 (2.6)
Ureteral stenosis	7 (1.7)
Isolated	4
With aponeurosis dehiscence	2
With lymphocele and incisional hernia	1
Venous thrombosis	7 (1.7)
Skin dehiscence	6 (1.5)
Lymphocele	6 (1.5)
Incisional hernia	6 (1.5)
Arterial thrombosis	1 (0.2)
Renal rupture	1 (0.2)
Ureteral leak treated with pyeloureterostomy (UL-PU)	n = 76
Total, n (%)	16 (21.0)
Urinary leak	7 (9.2)
Isolated	5
With skin dehiscence	1
With aponeurosis dehiscence	1
Ureteral stenosis	3 (3.9)
Surgical site infection	2 (2.6)
Skin dehiscence	2 (2.6)
Aponeurosis dehiscence	1 (1.3)
Lymphocele	1 (1.3)
Ureteral stenosis treated with pyeloureterostomy (US-PU)	n = 10
Total of complication	1 (10)
Ureteral restenosis	1 (10)

Seven patients (1.7%) developed pyeloureterostomy stenosis between 2 and 563 days of follow-up. Five patients (71.4%) received conservative treatment with double-J catheter replacement every 6 months. Of them, 2 (40%) developed recurrent urinary tract infections with acute renal dysfunction requiring hospital readmissions. One (14.3%) of these patients was submitted to a surgical correction, and the last one died due to urosepsis despite the use of culture-guided antibiotics and the location of a percutaneous nephrostomy (Table 2).

#### Pyeloureterostomy Secondary to Urinary Leak

Pyeloureterostomy was used to treat urinary leak (UL-PU) in 76 patients. Seven patients (9.2%) developed a recurrent urinary leak between 1 and 66 days after UL-PU, all successfully treated with subsequent surgical interventions. Patients were treated by a new pyeloureterostomy over a double-J catheter (n = 2), bladder suture of a previous Leadbetter-Politano ureterocystostomy (n = 2), suture at the leakage site (n = 1), and with nephrostomy (n = 1). The last patient was treated by a double-J catheter and indwelling vesical catheter insertion followed by protective nephrostomy and suture of the leakage area. All patients progressed with urinary fistulae resolution. Three patients (3.9%) developed ureteral stenosis between day 28 and 336 post-UL-PU, and all were treated conservatively with double-J catheter replacement every 6 months.

#### Pyeloureterostomy Secondary to Stenosis

Pyeloureterostomy (US-PU) was used in 10 patients with ureteral stenosis (8 Lich-Gregoir and 2 Leadbetter-Politano) following percutaneous nephrostomy (n = 4), retrograde placement of the double-J catheter (n = 4), or as a primary procedure (n = 2). One patient developed recurrent stenosis and was treated with double-J catheter replacement every 6 months.

#### Native Kidney Obstruction Requiring Nephrectomy

After a median follow-up of 33.8 months, ranging from 7 to 67 months, 10 patients (2%) required native nephrectomy (Table 3). Symptoms were lumbar pain with fever (n = 5) and isolated lumbar pain (n = 5). Among them, eight were patients in the P-PU, and 2 were in the UL-PU group.

**TABLE 3:** Native kidney nephrectomies after ureteral ligation for pyeloureterostomy.

Age (years)	Sex	CKD etiology	Residual diuresis (ml/day)	Type of surgery	Time after ureteral ligation (months)	Symptoms	Pathology	Outcome
59	Male	Diabetes Mellitus	0	P-PU	3	Fever	Hydronephrosis	Graft Nephrectomy/Death
12	Female	Neurogenic Bladder	500	UL–PU	5	Fever	Pyonephrosis	resolution
47	Male	Neurogenic Bladder	0	P–UP	4	Fever	Pyonephrosis	resolution
31	Male	Neurogenic Bladder	0	P–UP	16	Fever	Pyonephrosis	Graft Nephrectomy
55	Male	Diabetes Mellitus	0	P–UP	12	Fever	Pyonephrosis	resolution
54	Male	ADPKD	700	UL–PU	26	Lumbar pain	ADPKD	resolution
33	Male	Undetermined	500	P–UP	48	Lumbar pain	Hydronephrosis	resolution
47	Male	ADPKD	500	P–UP	11	Lumbar pain	ADPKD	resolution
50	Male	ADPKD	300	P–UP	19	Lumbar pain	ADPKD	resolution
48	Male	ADPKD	200	P–UP	13	Lumbar pain	ADPKD	resolution

CKD, chronic kidney disease; ADPKD, autosomal dominant polycystic kidney disease.

Of the five patients with fever, 3 (60%) had neurogenic bladder with prior bladder augmentation, and 2 (40%) had diabetes mellitus. The time between native ureter ligation and nephrectomy ranged from 3 to 16 months, and all but one patient had a final histological diagnosis of pyonephrosis. Two patients required graft nephrectomy due to associated infectious complications, and one of them subsequently died due to complications from an infected sacral ulcer. Five patients developed isolated lumbar pain 11–48 months after transplantation, and four of them (80%) had autosomal dominant polycystic kidney disease (ADPKD). All these patients showed favorable outcomes after the native nephrectomy.

Causes of native kidney nephrectomy were then hydronephrosis and pyonephrosis. Two demographic characteristics were associated with increased likelihood of native ipsilateral kidney complications requiring nephrectomy: ADPKD and augmented neurogenic bladder. In fact, the incidence of complications requiring nephrectomy was 13% among 31 patients with ADPKD (n = 4) and 27% among 11 patients with neurogenic bladder (n = 3).

## Discussion

Pyeloureterostomy is a well-known option for urinary tract reconstruction during kidney transplantation (3,4,8,24,26) as well as for the treatment of ureteroneocystostomy complications (10–13). At least two surgical techniques, end-to-end and end-to-side anastomosis, have been performed. Leadbetter *et al.* described the end-to-end reconstruction with native kidney nephrectomy in 1966 (26). Later, ipsilateral native nephrectomy was almost abandoned due to the low incidence of complications (6).

Despite the previous reports of the low incidence of major complications requiring nephrectomy, there are some concerns, mainly in those with more significant residual diuresis. For this reason, some surgeons advocate the use of end-to-side anastomosis to maintaining the urinary flow of the native kidney (27). Still, ureteral length and impaired endoscopic manipulation of the collecting system may offset the advantages of this surgical technique.

This single-center large cohort analysis revealed a low incidence (2%) of native ipsilateral kidney complications requiring nephrectomy in 495 kidney transplant recipients that underwent pyeloureterostomy without ipsilateral nephrectomy during the kidney transplantation or after urological complications. There were three graft losses (0.6%) and 2 deaths (0.4%) secondary to surgical complications.

A retrospective study including 278 kidney transplant recipients submitted to primary pyeloureterostomy with native ureter ligation without nephrectomy described an incidence of 2.2% (n = 6) of subsequent nephrectomy due to symptomatic hydronephrosis. Of these, 50% were in patients with chronic kidney disease due to ADPKD (6), findings similar to ours, in which 40% of the patients who underwent posterior nephrectomy had ADPKD. This increased risk is probably warranted by increased renal volume before the ureter ligation and more significant residual diuresis.

Guilter J *et al.* (25) observed a 3% incidence of native nephrectomy after ureter ligation and observed that high residual diuresis was a risk factor. However, in our study, this relation not observed since all the six patients who required posterior nephrectomy had less than 300 ml of urine output previously to the transplant, being four of them (80%) anuric.

One interesting observation of our cohort is that in patients who had previously undergone bladder augmentation, they had not only a higher risk of undergoing a future nephrectomy but also a more significant risk to unfavorable outcomes after the removal of their native kidney since 2 patients who had their nephrectomy indicated due to fever ended up losing their grafts, one of them dying soon after. We believe that colonization or infection of the urinary tract may predispose the occurrence of pyonephrosis in patients with hydronephrosis.

The urinary leak was diagnosed in 3.6% of patients after P-UP, an incidence similar to that reported in the literature (3–5%) for different urinary tract reconstruction techniques (2,16-19). On the other hand, in the UL-PU group, the incidence was 3 times higher (9.2%). A similar incidence (12.5%) was observed in other series (12), and this higher incidence is probably due to the inflammatory environment secondary to the urinary leakage. We chose to treat this complication according to the intraoperative findings, performing a new UP or locating a protective nephrostomy.

When the pyeloureterostomy anastomosis stenosis requires intervention, open correction using a surgical technique similar to that described by Anderson-Hynes for ureteropelvic junction (UPJ) stenosis may be considered. Yet, this procedure may be challenging due to the local hilar adherences. On the other hand, although the surgical risk associated with endourologic techniques low, the patency is approximately 60% after 5 years of follow-up (20–25). Given these caveats associated with both techniques, only one (10%) patient chose to undergo conventional surgical treatment while the remaining nine (90%) patients preferred periodic double-J replacement.

This analysis has limitations inherent to the retrospective nature of the study, potential selection bias in selecting the study population, and local surgical strategies that do not include the routine use of stenting for primary ureteroneocystostomy.

## Conclusion

End-to-end pyeloureterostomy with proximal ligation of the native ureter is a versatile procedure, allowing the reconstruction of the urinary tract even when the graft ureter is short, devascularized or when the recipient’s bladder is tiny and difficult to access. It is also an essential surgical technique to treat urinary leaks and stenosis, with complication rates similar to other types of reimplantation. The need for native nephrectomy was restricted to very few cases, occurring predominantly in patients with ADPKD and neurogenic augmented bladders, and was associated with low morbidity.

## Capsule Summary Sentence

This study aims to analyze the safety of the native ureter ligation without ipsilateral nephrectomy during pyeloureterostomy, used either as a primary surgical approach or as a secondary reconstructive technique after ureteral complications, in patients undergoing kidney transplantation.

## Data Availability

The raw data supporting the conclusions of this article will be made available by the authors, without undue reservation.
